# Atraumatic forearm swelling in a patient with poorly controlled asthma

**DOI:** 10.1016/j.rmcr.2021.101454

**Published:** 2021-07-01

**Authors:** Matthew Leon, Robert Liotta, Shambhu Aryal, Peter Vangeertruyden, Scott Tintle, Mary Klassen-Fischer, Aaron Holley, William Kelly, Jacob Collen

**Affiliations:** a Uniformed Services University of the Health Sciences, Bethesda, MD, USA; b Thoracic Radiology, Uniformed Services of the Health Sciences, Bethesda, MD, USA; c Musculoskeletal Radiology, Fort Belvoir Community Hospital, Fort Belvoir, Virginia, USA; d Hand Surgery, Department of Orthopedic Surgery, Walter Reed National Military Medical Center, Bethesda, MD, USA; e Thoracic Pathology, Joint Pathology Center, Silver Spring, MD, USA; f Pulmonary, Critical Care and Sleep Medicine, Walter Reed National Military Medical Center, Bethesda, MD, USA; g Advanced Lung Disease Program, Inova Fairfax Hospital, Falls Church, VA, USA; h Department of Medicine, Uniformed Services University of the Health Sciences, Bethesda, MD, USA

**Keywords:** Asthma, Bronchiectasis, Darier-Roussy disease, Extrapulmonary, Granulomatous disease, Sarcoidosis, Subcutaneous, BAL, bronchoalveolar lavage, CT, computed tomography, ED, emergency department, TBBX, transbronchial biopsy

## Abstract

We present a case of sarcoidosis presenting as unilateral forearm swelling. A 65-year-old male with a long history of asthma presented with unexplained left forearm and hand swelling. Over many years, chest imaging had been devoid of adenopathy or parenchymal findings suspicious for sarcoid, until after the extremity findings emerged. The patient was diagnosed based on subcutaneous, dermal and mediastinal lymph node histopathology. Sarcoid presenting with isolated extremity findings prior to more typical pulmonary manifestations is rare even for cutaneous or soft tissue sarcoid, highlighting the need to maintain a high index of suspicion for sarcoidosis.

## 1 Introduction

Sarcoidosis is a chronic, immune-mediated disease characterized by the presence of non-caseating granulomas. While the exact cause is unknown, it has been hypothesized that pathogens, environmental agents and chemical exposures may serve as triggers for the immune cell-mediated response [1]. Family/twin studies have pointed toward a genetic component to the disease with significant evidence of familial clustering [2,3]. Sarcoidosis is a multi-system disease with pulmonary involvement being most common [4]. Initial presentation without lung involvement is uncommon. In the seminal ACCESS study completed in 2001 only 2% of patients with sarcoid had single-system involvement outside of the lungs [5]. Subcutaneous sarcoidosis, a rare subset of cutaneous sarcoidosis, is a form of extra-pulmonary sarcoid commonly referred to as Darier-Roussy Disease [6]. It is typically characterized by asymmetric, bilateral nodules in the subcutaneous space of the torso and/or extremities without significant change to the overlying skin [6,7]. We report the case of a 65-year-old Caucasian male with obstructive lung disease presenting with isolated, unilateral, subcutaneous, non-caseating granulomas in the left forearm. His case has several atypical features which may further our understanding of how to screen for and treat individuals with this rare variant of sarcoidosis.

## 2 Case presentation

A 65-year-old nonsmoker veteran with a long history of asthma was referred by his primary care provider for follow-up. He had been experiencing monthly bouts of wheezing, dyspnea and productive cough, which resolve with a 5-day course of azithromycin and prednisone 50mg daily. In between bouts he was asymptomatic and managed his symptoms with budesonide and formoterol and as needed short acting albuterol. He had multiple surgeries for severe sinusitis and nasal polyps and experienced bouts of sinusitis treated with antibiotics three times per year and with nasal saline and steroid rinses daily. He had serially elevated serum immunoglobulin (Ig) E levels (several hundred IU/mL) and no peripheral eosinophilia. Biopsies from his sinus surgeries demonstrated chronic inflammation but no specific pathologic diagnosis.

Serial CT imaging of the chest over several years had demonstrated subtle waxing and waning peripheral tree and bud opacities without pathologic hilar or mediastinal adenopathy. A bronchoscopy was performed 3 years ago at another hospital for his CT findings with negative culture results and unremarkable endobronchial and transbronchial biopsy histology findings. His spirometry has been stable during several years of follow up with moderately severe obstruction and intermittent positive bronchodilator response. During flares he presented with expiratory wheeze on exam and more severe obstruction with a bronchodilator response, all of which improved with treatment.

Sputum cultures have been negative for microorganisms (including non-tuberculous mycobacterium) on several occasions, and his sinopulmonary flares diminished (every 2–3 months instead of monthly) on maximal medical therapy for asthma including budesonide/formoterol, tiotropium, as needed albuterol, montelukast, nasal fluticasone and omalizumab. He had no occupational, hobby, or animal exposures. On numerous occasions of the decade prior to this presentation, the patient had undergone serologic testing to evaluate allergic bronchopulmonary aspergillosis (ABPA), connective tissue disorders and hyper-eosinophilic syndromes. These tests had always been unremarkable (normal). Military service requires periodic mandatory screening for human immunodeficiency virus (HIV) and this patient was HIV negative. Complete blood counts with differential were normal (no peripheral eosinophilia) as well as metabolic panels (no hypercalcemia, renal or hepatic dysfunction). Laboratory studies were negative for Aspergillus fumigatus specific IgE (normal, 0.49 kU/L) and Aspergillus species antibody (normal/negative). Serum IgE values were in a non-specific range consistent with allergic asthma (134–840 IU/mL, over many years, never approaching 1000 IU/mL). The patient had normal levels of IgA, IgA and IgM.

It was also noted that he traveled several times per month around the mid-Atlantic region and occasionally overseas for business. After one plane flight he called his physician to report new atraumatic, painless swelling of the left forearm. He was instructed to go to the ER where doppler venous ultrasound was negative for deep vein thrombosis and his evaluation was otherwise normal.

**Fig. 1:**
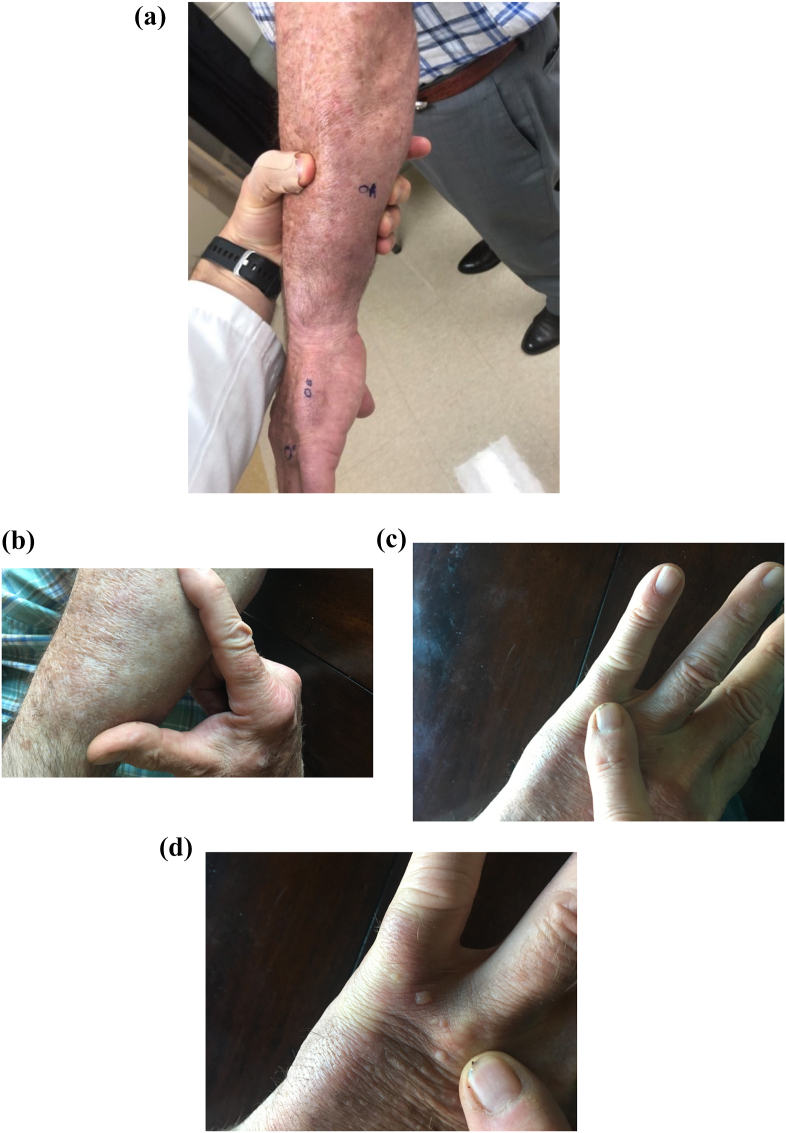
**a-d**: Photos of the external presenting appearance of skin abnormalities on the left forearm and hand: Fig. 1a and b: the left forearm with mild swelling (non-pitted, non-indurated, no erythema); 1b (close view). Fig. 1c and d: there are some subtle irregular papules on the dorsum of the left hand without discoloration (1d), without digital nail pitting or clubbing (1c).

**Fig. 2:**
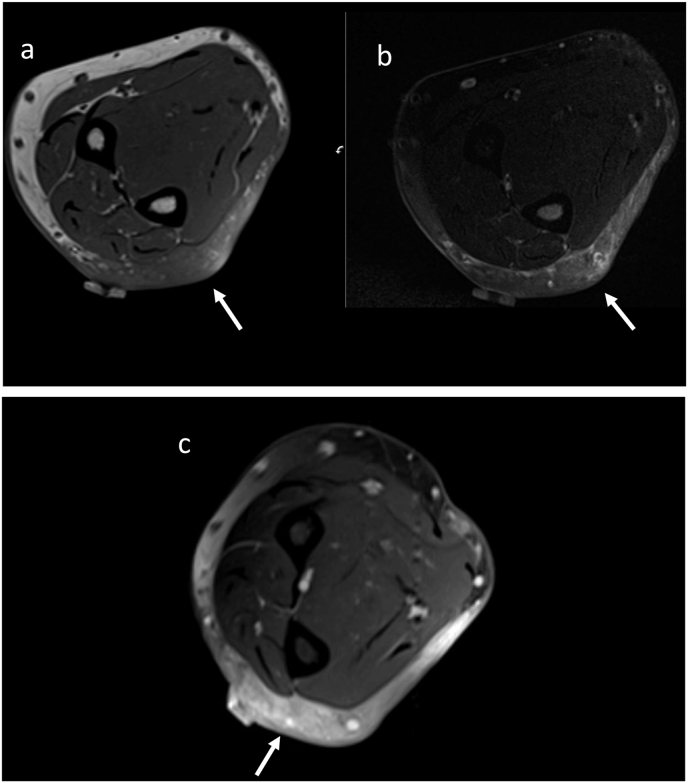
a, b, c: **(a)** Axial T1-weighted MRI image demonstrates diffuse low signal non mass like infiltration of the subcutaneous tissues of the posterior elbow (arrow). **(b)** Axial fat saturated T2 image shows areas of increased reticular signal within the posterior elbow subcutaneous tissues (arrow). **(c)** Axial contrast enhanced fat-saturated T1-weighted MRI image demonstrates diffuse non mass like enhancement of the subcutaneous tissues of the posterior elbow (arrow).

**Fig. 3:**
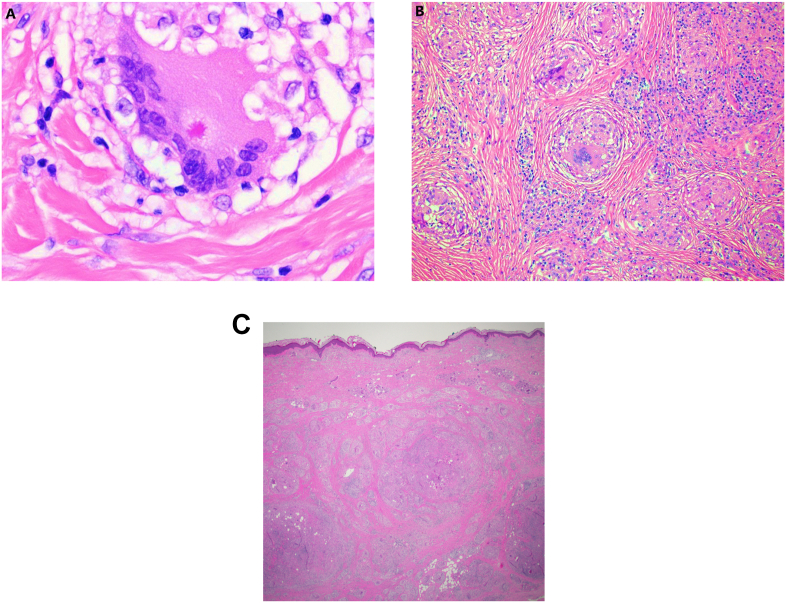
**a, b, c**: **(a)** Multinucleated giant cell containing an asteroid body, which may be seen in granulomas of any etiology (H&E, original magnification 600x). **(b)** Higher power view showing multinucleated giant cells within a granuloma. (H&E, original magnification 100x). **(c)** Low power view of the skin biopsy showing that the dermis is heavily infiltrated by round non-necrotizing granulomas (H&E, original magnification 12.5x).

At follow-up in clinic a week later the patient's forearm had progressed to multiple lesions that were firm, non-tender, and palpable in the subcutaneous tissue with no outward appearance on the skin or digital clubbing or pitting of the nails (Fig. 1a, b, 1c, 1d). One month after the onset of his left arm swelling in February 2019, an MRI of the arm revealed nonspecific edema, fibrosis, and heterogeneous enhancement. No focal mass or abnormal fluid collection was visualized. (Fig. 2a, b, 2c). An open surgical biopsy was performed by orthopedics and a skin biopsy was performed by dermatology (Fig. 3a, b, 3c).

**Fig. 4:**
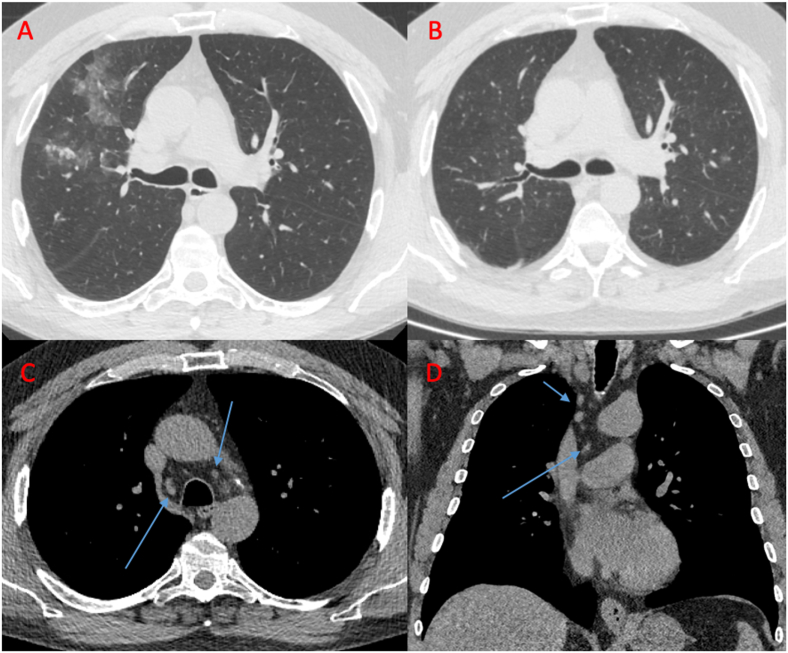
a, b, c, d: CT images obtained in 2011, 2015, and 2019. **(a)** Axial CT image in December 2011 with patchy centrilobular ground-glass attenuation and tree-in-bud nodularity involving the right upper lobe. **(b)** Axial CT image in October 2015 with only minimal scattered centrilobular ground-glass attenuation and nodularity involving both upper lobes. **(c, d)** Axial and Coronal CT images in January 2019 show small mediastinal and paratracheal lymph nodes (arrows) which are not pathologic based on size criteria (all less than 10 mm in short axis diameter) and unchanged from prior exams.

**Fig. 5:**
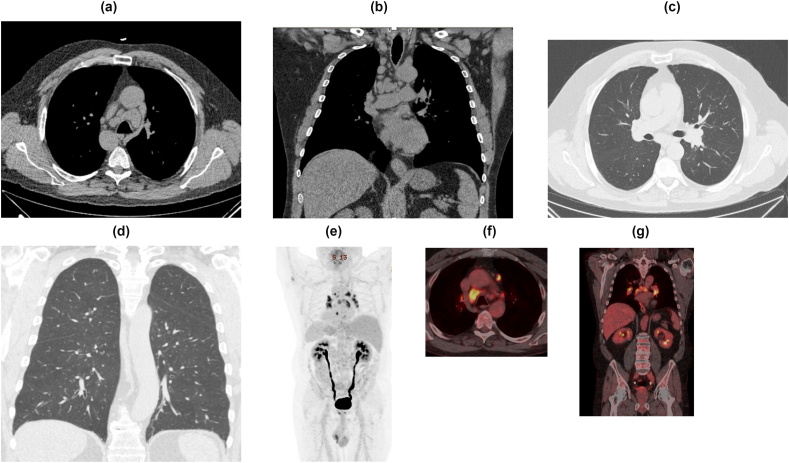
a, b, c, d, e, f, g: CT images obtained in July 2019. **(a, b):** Axial and Coronal CT images show numerous enlarged mediastinal and paratracheal lymph nodes (arrows). **(c, d):** Axial and Coronal CT images with lung window settings show normal lung parenchyma. **(e,f, g):** Corresponding PET-CT images with increased metabolic activity of the mediastinal and paratracheal lymph nodes.

In both cases, the results of the pathology reports demonstrated non-caseating granulomas in the dermis and subcutis (sparing the muscle). Prior to the biopsy results, sarcoidosis was considered but the available data was not sufficient to make the diagnosis. Although a CT scan done in January 2019 did not show adenopathy (Fig. 4a–d), a repeat scan in July 2019 (several weeks after the presentation discussed in this article), demonstrated new bulky bilateral hilar and mediastinal lymphadenopathy (Fig. 5a–d). Bronchoscopic biopsy at this time was performed with endobronchial ultrasound and demonstrated non-caseating granuloma. An F18-FDG PET/CT scan in August 2019 confirmed the presence of hypermetabolic mediastinal and left axillary lymphadenopathy, accompanied by hypermetabolic activity along the left forearm in the area where non-caseating granulomas had been biopsied (Fig. 5e,f,g). Angiotensin converting enzyme (ACE) testing at that time was unremarkable (two prior tests, < 20 U/L, normal 14–82 U/L).

Our patient was started on a 40 mg daily regimen of Prednisone for 3 months from September to November 2019. A repeat PET scan was performed, and results showed an interval decrease in the size and FDG avidity of the mediastinal lymph nodes. Despite the positive imaging findings, he was having difficulty tolerating the 40mg dose due to weight gain, swelling, and exacerbation of his preexisting GERD. The decision was made to add Methotrexate 10mg per week while tapering the prednisone. In January 2020, one week after tapering off the prednisone, the patient reported the arm lesions were worsening. He was placed back on Prednisone, this time at 20 mg, in conjunction with the methotrexate. This combined therapy helped stop the recurrent lesions in the arm and the patient reported continued and durable improvement in his respiratory symptoms after restarting the prednisone at a lower dose. One year following presentation the patient is on 20mg daily of methotrexate only with no recurrence of the arm or skin lesions and stable to slightly improved spirometry.

## 3 Discussion

This case raises several discussion points regarding the pathogenesis and diagnosis. In this case, the patient had a long history of obstructive pulmonary disease that was responsive to oral corticosteroids when administered in brief courses for symptomatic flares of disease. The consensus was that his years of pulmonary disease were the product of refractory asthma. Certainly, his PFTs supported this diagnosis with spirometry demonstrating moderate-to-severe reversible obstruction and expiratory wheezing that resolved in between bouts. Serial CT imaging of the thorax had failed to demonstrate lymphadenopathy or granulomatous disease. There was also no evidence in yearly (and more frequent) CT imaging of the thorax of interstitial lung disease progression. There were waxing-and-waning subtle tree-and-bud infiltrates and borderline findings that could be consistent with mild bronchiectasis developing in the base of the right lung. The patient had a long history of Barrett's esophagus and gastroesophageal reflux disease, with ongoing late meals and dependence on chewing tobacco, so these imaging findings were thought to represent chronic recurrent episodes of aspiration. In 2013, the patient underwent bronchoscopy and transbronchial and endobronchial biopsies, as well as needle biopsies of the hilum and carina with no evidence of granulomatous disease.

Although sarcoidosis is generally a restrictive disease there is significant literature documenting obstructive lung disease. The ACCESS study found that 46.9% of the patients had an FEV1/FVC ratio of greater than 80% compared to only 13.2% with an FEV1/FVC ratio between 50 and 69% [5]. More recent studies posited that the number of patients with sarcoidosis and obstructive lung function may be higher than what was previously documented. In a 10-year retrospective analysis of sarcoid patients presenting in their clinic, Thillai et al. found that the percentage of individuals with sarcoidosis and an obstructive lung picture (FEV1/FVC between 50 and 69%) was closer to 24% [8]. Obstructive PFTs were more common in older, white individuals with sarcoidosis, compared with black patients with sarcoidosis [8].

Ultimately, it was the new complaint of isolated upper extremity swelling and painless, atraumatic induration with overlying skin changes over a segment of the forearm that led to the diagnosis. Although the vast majority of sarcoid patients present with pulmonary findings, extrapulmonary sarcoid is not uncommon. Cutaneous sarcoid can present in 9–37% of patients and soft tissue sarcoidosis in 1.4–16% [4,7]. Generally, cutaneous sarcoid presents with painless nodules, papules and plaques. In cases of sarcoid that presents in the extremities, both upper and lower extremity presentations have been well documented, but usually multiple limbs are involved, and more diffuse distribution of skin findings are documented. We were able to find one citation documenting a case that presented with bilateral arm swelling alone [9]. The time course of our patient's presentation, history of asthma, numerous negative prior evaluations (imaging, serology, biopsy), eventually presenting with an atypical subcutaneous lesion in only one forearm, has not be reported previously. Fortunately, the patient had a brisk and durable response to anti-inflammatory therapy.

The patient's response to treatment in this case is also an important point for discussion. It is well established that corticosteroids are the 1st line treatment for symptomatic sarcoidosis and that tapering, when possible, is best practice to avoid the associated drug side effects [10]. However, the correct dosing of corticosteroids and the concurrent use of 2nd and 3rd line treatments is still debated [10]. Our case demonstrates the use of methotrexate to maintain the treatment benefits garnered from the initial course of prednisone while minimizing side effects, which is an established clinical practice for sarcoidosis.

## 4 Conclusion

Although sarcoidosis is a common disorder, it is important to remember that this disease can present without hallmark symptoms and in atypical disease locations. Presentation isolated to a single upper extremity is exceedingly rare. Steroid therapy failure should inspire reflection but does not rule out sarcoidosis. Our case highlights the need to remain vigilant for mimics of asthma, including sarcoidosis, eosinophilic granulomatosis with polyangiitis (Churg-Strauss syndrome), allergic bronchopulmonary aspergillosis (ABPA), hypersensitivity pneumonitis, bronchiectasis and interstitial lung disease. Tissue sampling and histology are still often a critical piece of solving the puzzle. Fortunately, research in the diagnosis and treatment of sarcoid remains active and society guidelines for both diagnosis and treatment have recently been published [11,12]. Close follow-up over time is key, as the presentation of inflammatory disorders can be misleading initially and evolve over time. This is especially important to remember in healthcare systems where the treating physician may change over time due to staff turnover.

## Declaration of competing interest

The authors have no conflicts of interest to disclose.
